# Hands-On Defibrillation Skills of Pediatric Acute Care Providers During a Simulated Ventricular Fibrillation Cardiac Arrest Scenario

**DOI:** 10.3389/fped.2018.00107

**Published:** 2018-04-23

**Authors:** Utpal S. Bhalala, Niveditha Balakumar, Maria Zamora, Elumalai Appachi

**Affiliations:** ^1^Pediatrics, Baylor College of Medicine, Houston, TX, United States; ^2^Pediatric Critical Care Medicine, Pediatrics, The Children's Hospital of San Antonio, San Antonio, TX, United States; ^3^Pediatrics, Miami Children's Hospital, Miami, FL, United States

**Keywords:** defibrillation, hands-on skills, simulation, defibrillators, ventricular fibrillation, cardiac arrest, CPR

## Abstract

**Introduction:** Timely defibrillation in ventricular fibrillation cardiac arrest (VFCA) is associated with good outcome. While defibrillation skills of pediatric providers have been reported to be poor, the factors related to poor hands-on defibrillation skills of pediatric providers are largely unknown. The aim of our study was to evaluate delay in individual steps of the defibrillation and human and non-human factors associated with poor hands-on defibrillation skills among pediatric acute care providers during a simulated VFCA scenario.

**Methods:** We conducted a prospective observational study of video evaluation of hands-on defibrillation skills of pediatric providers in a simulated VFCA in our children's hospital. Each provider was asked to use pads followed by paddles to provide 2 J/kg shock to an infant mannequin in VFCA. The hands-on skills were evaluated for struggle with any step of defibrillation, defined a priori as >10 s delay with particular step. The data was analyzed using chi-square test with significant *p*-value < 0.05.

**Results:** A total of 68 acute care providers were evaluated. Median time to first shock was 97 s (IQR: 60–122.5 s) and did not correlate with provider factors, except previous experience with the defibrillator used in study. The number of providers who struggled (>10 s delay) with each of connecting the pads/paddles to the device, using pads/paddles on the mannequin and using buttons on the machine was 34 (50%), 26 (38%), and 31 (46%), respectively.

**Conclusions:** The defibrillation skills of providers in a tertiary care children's hospital are poor. Both human and machine-related factors are associated with delay in defibrillation. Prior use of the study defibrillator is associated with a significantly shorter time-to-first shock as compared to prior use of any other defibrillator or no prior use of any defibrillator.

## Introduction

Approximately 450,000 Americans have cardiac arrest annually [1]. As opposed to previous reports, recent studies suggest improved survival to discharge among pediatric, in-hospital cardiac arrests, driven largely by an improvement in resuscitation techniques [2–4]. The prevalence of ventricular fibrillation (VF) or pulseless ventricular tachycardia (PVT) as the first documented rhythm has been shown to be 14% in children [3]. For VF and PVT arrest, prompt defibrillation and high-quality CPR are recommended. Despite improved understanding of pathophysiology of cardiac arrest and impact of quality CPR on survival from arrest, there is sufficient evidence to suggest that most components of CPR, including time-to-first shock in VFCA do not meet quality standards [5–9]. For every minute delay in defibrillation, survival rates from witnessed VFCA decrease 7–10% and with CPR, the decrease in survival averages 3–4% per minute [10].

There is lack of sufficient data to suggest if providers in pediatric acute care settings are deficient in specific steps of hands-on defibrillation skills. Also, the factors associated with their poor defibrillation skills are largely unknown. Basic and advanced life support courses neither provide mastery with hands-on CPR skills nor do they assist with retention of skills [11, 12]. Also, particularly in pediatrics, we are challenged with the issue of paradox—the skills needed to acutely save a child's life are also the skills we get to perform and practice the least [13]. Different simulation-based training interventions, including rapid cycle deliberate practice has shown to improve defibrillation skills of pediatric residents [14, 15]. Improving our understanding of individual steps of the defibrillation will allow us recognize any pattern and improve existing simulation-based defibrillation skill curricula, particularly deliberate practice-based. We aimed at evaluating delay in individual steps of the defibrillation and human and non-human factors associated with poor hands-on defibrillation skills among pediatric acute care providers during a simulated VFCA scenario.

## Methods

### Study design

We conducted a prospective observational study of hands-on defibrillation skills of pediatric acute care providers in a simulated VFCA in our children's hospital. The institutional review board of Baylor College of Medicine and feasibility committee of Voelcker Clinical Research Center of The Children's Hospital of San Antonio approved the study.

### Primary outcome measure

The primary outcome measure was time-to-first-shock defined a priori as time elapsed between switching on the defibrillator until successful delivery of the shock. Successful delivery of the shock was defined as appropriately pressing the “shock” button on the defibrillator while using the pads on the mannequin or both the “shock” buttons on the paddles while using the paddles on the mannequin.

### Secondary outcome measures

Secondary outcome measures included video assessment of factors associated with delay in defibrillation and delay in different steps of defibrillation. The different steps of defibrillation which were objectively assessed for delay by reviewing videos included applying pads and paddles to mannequin, connecting pads and paddles to the device, turning the device on, selecting energy, charging the device and applying shock.

### Study population

The physicians, nurses and nurse practitioners either working or rotating in our pediatric intensive care unit (PICU) and pediatric emergency room (ER) were recruited for the study. A written informed consent was obtained from each study subject. In order to minimize selection bias, we recruited subjects during day and night shifts of weekdays and weekends. In order to avoid any bias which could potentially occur due to participation in a recent resuscitation effort before actually participating in the study, we recruited most of the subjects during randomly selected shifts of weekdays and weekends over a short span of 15 days and asked each subject to sign a confidentiality agreement while signing the consent form. “The subjects were recruited during randomly selected shifts when UB was not working in the ICU and available to recruit the subjects.”

### Baseline characteristic survey

In the beginning of the study, each study participant was asked to fill out demographic data on type of provider, training level, years in practice, primary location of work, basic and advanced life support certification details and information on their experience with the use of defibrillator/s.

### Simulated ventricular fibrillation cardiac arrest (VFCA)

**Figure 1:**
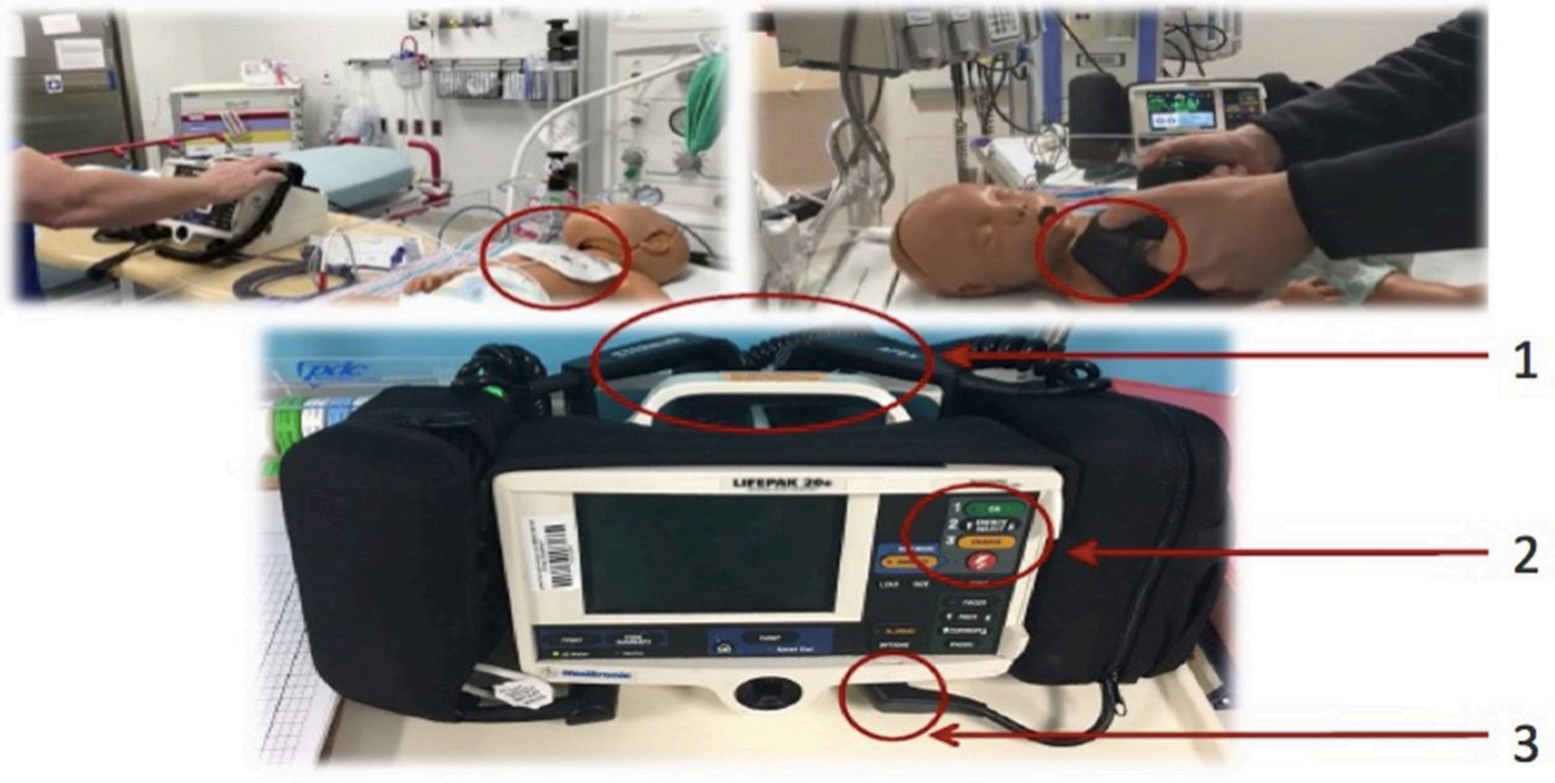
The top left picture shows a pediatric provider using pads on the pediatric mannequin, whereas the top right picture shows a pediatric provider using paddles on the pediatric mannequin. Note use of adult paddles instead of pediatric paddles in the top right picture. The bottom picture shows LifePak 20e (study) defibrillator. A 10 s or longer delay in using either pads/paddles (1) or buttons (2) or cords (3) was considered a “struggle” with the specific step of defibrillation.

The participant was described the case scenario^*^ and asked to provide defibrillation shock to the infant mannequin using defibrillation pads followed by repeating the entire scenario using the defibrillation paddles. The participants' hands-on defibrillation skills which were assessed through the review of video file of the scenario included manually connecting the pads/paddles to the mannequin at one end and to the device at the other end, turning the device on, selecting energy, charging the device and applying shock using either the shock button on the device while using pads or the shock buttons located on the paddles while using paddles. Since the primary study focus was providers' hands-on defibrillation skills and not necessarily their ability to identify rhythm on the monitor or the appropriate dose of energy, we, at the beginning of the scenario gave away the information about VF rhythm on the monitor and the energy selection for the defibrillation shock to the participants. Also, since the focus of the study was not necessarily evaluating the choreography of high-quality chest compressions in relation to the defibrillation, the participants were instructed that another provider already started chest compressions as soon as VFCA was identified on the monitor. The defibrillator used in our study was LifePak 20e (“Study defibrillator”) (Physio-control Inc., Redmond, WA, USA). In our institution, all the defibrillators are located on the code cart with a set of pads and electrodes stored in the side pouches of the defibrillator (Figure 1). In order to keep the process of defibrillation in our study close to “real” and to maintain consistency with each scenario, we provided the defibrillator the way it is always kept on our code cart (Figure 1). The VFCA scenario and therefore hands-on defibrillation skills assessment ended when the participant successfully applied shock using shock button on the device while using pads or shock buttons on the paddles while using paddles. Though the use of paddles in most institutions, including ours, has significantly decreased over time, paddles being an integral part of the defibrillators, could be helpful in those situations in which pads may not be readily available. We therefore decided to include evaluation of hands-on defibrillation skills of the study participants using paddles.

### Data abstraction

**Figure 2:**
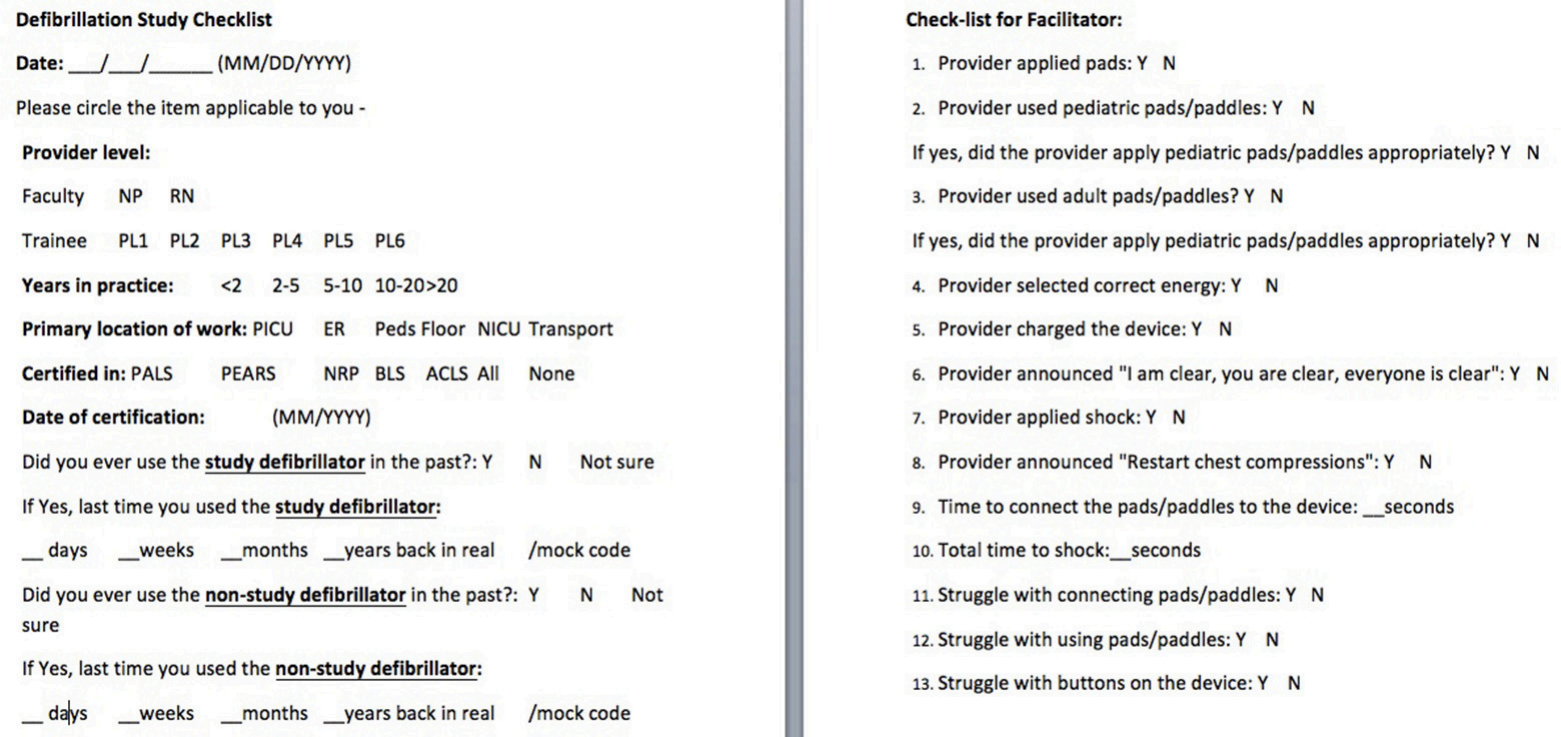
The defibrillation Study Checklist showing the provider-specific questions and defibrillation-specific questions.

Each scenario was captured on a video and video files were transferred to and stored in a secured, password protected, institutional file sharing tool called Baylor College of Medicine Box (Box, Inc., Redwood City, CA, USA). Separate video files were created for defibrillation performed using pads and paddles. Two independent reviewers, NB and EA evaluated each video file and moderator UB resolved any disagreement on the findings of the 2 reviewers. During video evaluation of each scenario, the reviewers used “Defibrillation Study Check-list” to evaluate for different components of defibrillation (see Figure 2). Conventionally, the time-to-first-shock is defined as the time the rhythm is first identified as shockable rhythm to the actual delivery of the shock [5, 6]. Since the primary study question was evaluation of providers' hands-on defibrillation skills and not their ability to identify the rhythm, the time-to-first-shock in our study was defined a priori as time the defibrillator was switched on by the subject to the actual delivery of the shock. Since one of the main objectives of the study was to evaluate machine-related factors associated with poor hands-on defibrillation skills, the reviewers were asked to assess for provider's “struggle” using the machine. The “struggle” with machine was defined a priori as ≥ 10 s delay in individual machine components. We attempted to evaluate for potential reasons for struggle with using individual components of the machine. The individual components of the defibrillation process which were analyzed by the reviewers were connecting the pads/paddles to the mannequin at one end and to the device at the other end, turning the device on, selecting energy, charging the device and applying shock using either the shock button on the device while using pads or the shock buttons located on the paddles while using paddles. We also evaluated the use of adult instead of pediatric paddles and pads and appropriate placement of paddles and pads on the mannequin. Data were entered into a Microsoft Excel® database and analyzed using Stata 10 (StataCorp, College Station, TX).

### Statistical analysis

Analyses were conducted for baseline characteristics and outcome variables, stratified by provider category (nurse, non-nurse). For categorical variables, proportions were compared using the chi-square test. The variables not normally distributed were reported in median with interquartile range (IQR). Analysis was performed to determine any statistically significant effect modification on the outcome between the following variables, which were chosen a priori: “Category” (nurse or non-nurse), “Years of experience” (<5 Yr or >5 Yr), “Primary site of work” (PICU or non-PICU), “PALS training” (<3 Yr or >3 Yr), “prior use of defibrillator” (study defibrillator or any other defibrillator).

^*^VFCA Case Scenario: A 2 month old baby boy who is brought to the ER/PICU for URI symptoms and worsening respiratory distress develops ventricular fibrillation and you are asked to provide first defibrillation shock while CPR is on-going. This is the defibrillator machine available to you. Please go ahead and provide 2 J/kg/dose of defibrillator shock to this patient. The patient's weight is 10 Kg.

## Results

**Figure 3:**
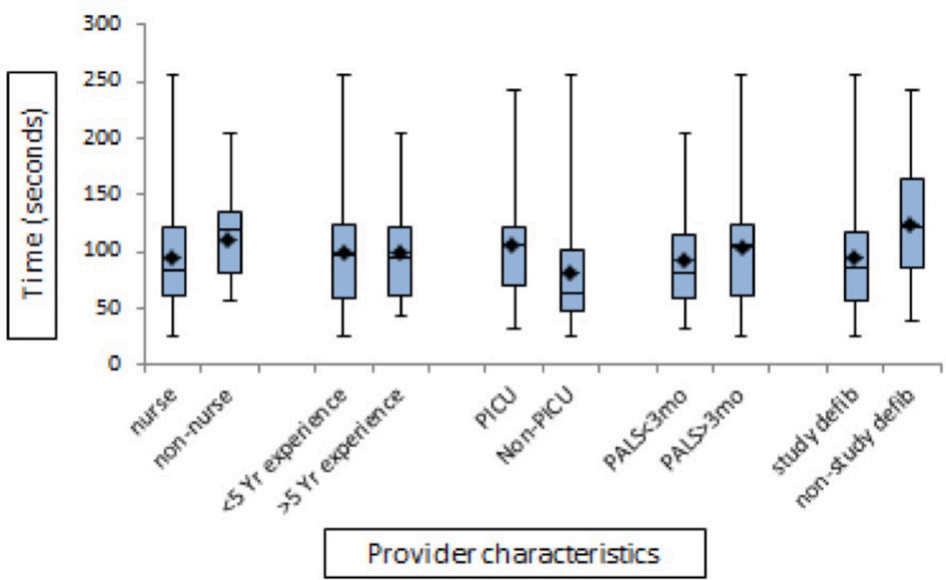
The bar diagram showing comparison of provider characteristics and time to shock in seconds with use of defibrillator paddles.

A total 68 acute care providers (50 nurses and 18 non-nurse providers) were evaluated for time-to-first shock using the study defibrillator. Of the 68 subjects, 46% (31/68) had <5 years and the remaining had >5 years experience in acute care setting. Majority (50/68) of subjects were PICU providers, while the remaining were ER providers. All the subjects were PALS certified and the proportion of providers additionally certified in PEARS, NRP, ACLS and BLS was 4% (3/68), 10% (7/68), 61% (42/68), and 73% (50/68) respectively. The median time elapsed between the day of last PALS certification and the day the defibrillation skills were tested was 273 days (IQR: 90–395 days). The proportion of providers who had previously used the study defibrillator was 79% (54/68). Of these, only 14% (8/54) had used the study defibrillator in real scenario whereas 81% (44/54) had used it in mock scenario. The median time elapsed between the prior use of the study defibrillator and the day of study was 90 days. The proportion of providers who had previously used a defibrillator other than LifePak 20e prior to the study was 39% (27/68). Of these, 48% (13/27) had used the defibrillator in real scenario whereas 51% (14/27) had used it in mock scenario. The median time-to-shock was 77.5 (IQR: 59–105) s. with the use of pads and 97 (IQR: 60–122) s. with the use of paddles. There was no significant difference in time-to-first shock between nurse vs. non-nurse, <5 Yr vs. >5 Yr experienced and PICU vs. Non-PICU, recent (<3 mo) PALS-trained vs. non-recent (>3 mo) PALS-trained providers (*p* > 0.05). There was a significant difference between those who had used the study defibrillator as compared to those who had never used it in the past for time-to-first shock (*p*-value 0.047) (Figure 3).

**Figure 4:**
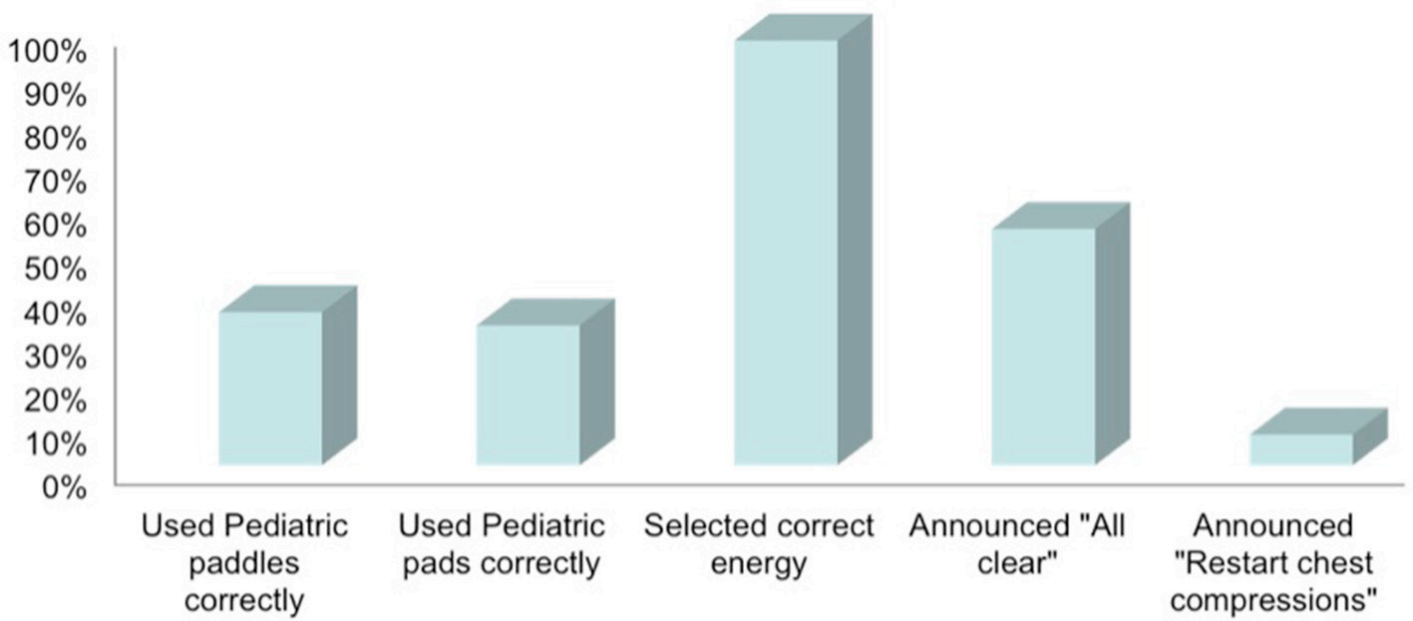
Bar diagram-showing percentage of providers who met quality and safety defibrillation standards.

The proportion of providers who struggled (>10 s delay) with connecting the pads/paddles to the device was 50% (34/68). The struggle with connecting pads/paddles to the device was observed to be related to the presence of multiple electrode wires and cords in different part of the defibrillator, difficulty with locating the socket where pads/paddles would connect to the defibrillator if they were not already connected to the defibrillator or if the pads needed to be switched to paddles and vice versa, difficulty with inserting the plug of the pads/paddles into the defibrillator socket. All the providers struggled with twisting and un-twisting the plug of the pads/paddles to disconnect it from the socket of the defibrillator. This step was required while switching pads from paddles and vice versa. The proportion of providers who struggled with applying the pads/paddles to the mannequin was 38% (26/68). The struggle was observed to be related to appropriate placement of very big adult-size paddles and pads on the infant mannequin. The proportion of providers who struggled using buttons on the device was 46% (31/68). The struggle with using buttons on the defibrillator was observed to be related to struggle with identifying buttons for changing the energy, charging the device and applying shock and presence of same shaped buttons for different functions arranged one below the other on the machine. Some delay was also observed to be related to use of “manual” button in the beginning for either changing energy or charging the device. Also, during the use of paddles, 90% (61/68) providers either struggled with or did not use “charge” and “shock” buttons which are built into the paddles for convenience. Prior use of the same defibrillator in real/mock scenario did not reduce the chance of any struggle with defibrillation. Only 35% (24/68) providers used pediatric paddles and out of these 24 providers who used pediatric paddles, 100% had struggle with removing the large adult paddles to convert to pediatric paddles. Only 32% (22/68) providers applied the paddles/pads correctly on the mannequin. The remaining 46 providers either applied pads/paddles touching each other or not at appropriate places to allow optimum energy to pass through the heart. Only 54% (37/68) providers announced, “I am clear, you are clear, everybody is clear” before delivering the shock and only 7% (5/68) providers announced to re-start the chest compressions soon after the shock was delivered (Figure 4).

## Discussion

During VFCA, timely and effective defibrillation is key to a successful outcome. To test the ability to provide a timely shock in a VFCA scenario, we studied time-to-first shock, defined a priori as time elapsed between switching the machine on to actual provision of shock. To test the ability to provide effective shock, we studied parameters such as selection of correct energy and appropriate application of the pads/paddles on the mannequin. Previous studies evaluated defibrillation times of resident physicians [7, 14]. None of these studies reported or compared the defibrillation times of faculty and nurses. In majority of in-hospital VFCA scenario, the physicians lead the code and a nurse or other physician operates the defibrillator. Therefore our study of defibrillation skills of physicians and nurses is practically more relevant study as compared to the previous studies of defibrillation skills of resident physicians. In the study by Hunt et al., the median times to notice and recognize shockable rhythm were 3–4.5 s and 10–26 s respectively, whereas the median time to ask for defibrillator was 20–40 s among pediatric residents [7]. If we incorporate the time taken each for recognizing and confirming shockable rhythm and time taken to bring the defibrillator to the victim, the median time of 77.5–97 s to operate the defibrillator and apply appropriate shock found in our study would not comply with the AHA guidelines [10]. In short, our findings support the findings of delayed defibrillation reported in the previous studies.

To analyze the provider-related factors associated with delayed defibrillation, we compared provider variables for the time-to-first shock. Only the prior use of the study defibrillator was associated with a statistically significantly shorter time-to-first shock as compared to either prior use of a defibrillator other than study defibrillator (Lifepak 20e) or no prior use of any defibrillator. This observation is somewhat different from the findings of the prior study in which any use of defibrillator was 87% more likely to be associated with successful defibrillation [7]. The findings of our study further suggest that since there are obvious differences in different models of defibrillators, hands-on training on one particular model does not necessarily guarantee mastery on hands-on skills with another model. Contrary to the findings of previous studies, our study had a much higher proportion [45% (31/68)] of providers who did not announce, “I am clear, you are clear, everybody is clear.” This could have been related to the fact that in this simulated scenario, the providers were instructed to focus on the defibrillation task and that there were no other providers physically present at the scenario carrying out tasks such as compressions or ventilation.

Given that 27% of pediatric patients who have an in-hospital cardiac arrest will have a shockable rhythm at some point, we must ensure nurses, physicians and nurse practitioners in pediatric acute care settings can appropriately defibrillate on time [3]. The findings of our study suggest that neither the provider category and experience, nor the certification status is associated with superior defibrillation skills. In PALS and ACLS course contents, testing the hands-on defibrillation skills of the course participants is not an explicit requirement of these courses [16, 17]. The findings of our study and previous similar studies suggest a need for incorporating hands-on defibrillation skills training and testing within PALS and ACLS course contents. In certain high-risk industries such as automotive and aviation, acquisition of a license to “drive” or “fly” is mandatory and a “drive test” or a “fly test” is often a requirement. Our healthcare industry is high-risk and high-stake industry but neither a license to “defibrillate” nor a “defibrillation test” has been mandated yet! Hands-on training is important for safe and efficacious use of any system/process, which involves human-machine interface. It is about time to consider rigorous hands-on defibrillation skills curricula with frequent hands-on defibrillation training in our healthcare industry.

To our knowledge, this is the first study which has evaluated the factors related to the delay in defibrillation by nurses, physicians and nurse practitioners of a tertiary care children's hospital. The prior studies on delay in defibrillation in a simulated CA describe similar issues of “struggle” with the machine but our study, for the first time provides quantitative assessment of how different components of the machine contribute to the delay in defibrillation. The defibrillators need to minimize clutters of wires and cords with appropriate, visually appealing labels to easily and rapidly differentiate wires and cords. The defibrillator buttons should be designed and located on the machines such that they minimize “amygdala hijack” and help the providers with easy and rapid “switch on,” selection of the energy, charging of the device and application of the shock.

Our study findings will help to develop hands on defibrillation skill curricula, which are focused on individual steps of defibrillation. The information will also help the industry partners create and test more user-friendly defibrillator prototypes which could potentially minimize if not eliminate the machine-related factors associated with delay in defibrillation.

## Limitations

Our study has several limitations. We did not account for time to identify the shockable rhythm or time to get the defibrillator to the mannequin, but since the focus was hands-on defibrillation skills, we analyzed the time-to-operate defibrillator and deliver shock. Because of the distribution of nurses and non-nurse providers in our children's hospital, the study population was skewed to more nurse participants as compared to non-nurse participants. Our study did not compare the hands on defibrillation skills using different defibrillator models. Since there is only one particular model of defibrillator in our children's hospital, it made sense to study the model, which would be available to us in real VFCA scenario. In our study, review of videos for the delays with individual components of defibrillation allowed us understand possible reasons for such delays. For example, we observed that the buttons for charging the device, changing the energy and applying shock were of same size and shape and located one above the other and therefore responsible for delay in using these buttons, but we did not corroborate these observations through a post-scenario questionnaire of the participants for their perspective. Lastly, this was a study of simulated VFCA with inherent simulation-related limitations. For example, since the providers were instructed to focus on the defibrillation and that there were no other providers physically present at the scenario to provide chest compressions or bag-mask ventilation, we believe that it might have contributed to a large proportion of providers not announcing “I am clear, you are clear, everybody is clear” before the delivery of shock.

## Conclusions

The defibrillation skills of physicians, nurses and nurse practitioners in a tertiary care children's hospital are poor. Both human and machine-related factors are associated with delay in defibrillation. Prior use of the study defibrillator is associated with a significantly shorter time-to-first shock as compared to prior use of defibrillator other than study defibrillator or no prior use of any defibrillator. Each institution should design and implement hands on defibrillation skills curriculum, especially using the defibrillator which is used in the respective institution. The industries should come up with solutions to create user-friendly defibrillators.

## Author contributions

All the authors contributed equally to the research project and the manuscript.

### Conflict of interest statement

The authors declare that the research was conducted in the absence of any commercial or financial relationships that could be construed as a potential conflict of interest.
